# Glymphatic system dysfunction in patients with early chronic kidney disease

**DOI:** 10.3389/fneur.2022.976089

**Published:** 2022-08-08

**Authors:** Chang Min Heo, Dong Ah Lee, Kang Min Park, Yoo Jin Lee, Sihyung Park, Yang Wook Kim, Junghae Ko, Byeong Cheol Yoo, Bong Soo Park

**Affiliations:** ^1^Departments of Internal Medicine, Haeundae Paik Hospital, Inje University College of Medicine, Busan, South Korea; ^2^Departments of Neurology, Haeundae Paik Hospital, Inje University College of Medicine, Busan, South Korea; ^3^Department of Clinical Research, DEEPNOID, Seoul, South Korea

**Keywords:** glymphatic system, diffusion tensor imaging, chronic kidney disease, DTI-ALPS index, neurological complication

## Abstract

**Introduction:**

It is a recent finding that glymphatic system dysfunction contributes to various neurological problems. The purpose of this research was to assess the function of the glymphatic system in neurologically asymptomatic early chronic kidney disease (CKD) patients and healthy controls, using diffusion tensor image analysis along perivascular space (DTI-ALPS) index.

**Methods:**

In a prospective study, we included patients with early CKD who were asymptomatic for neurological issues and obtained clinical and laboratory data. In all participants, brain magnetic resonance imaging (MRI) with diffusion tensor imaging (DTI) was conducted. We used DSI program for DTI preprocessing and DTI-ALPS index estimation. The DTI-ALPS index was compared between patients with early CKD and healthy controls, and the association between clinical characteristics and the DTI-ALPS index was investigated.

**Results:**

Eighteen patients with early CKD and 18 healthy controls were included in this study. Patients with early CKD had lower DTI-ALPS index than healthy controls (1.259 ± 0.199 vs. 1.477 ± 0.232, *p* = 0.004). In the correlation analysis, the DTI-ALPS index had no significant relationship with other clinical factors.

**Conclusion:**

We suggest dysfunction of glymphatic system in patients with early chronic kidney disease using the DTI-ALPS index. This may be related to the pathophysiology of neurological problems including impairment of cognition in patients with early CKD.

## Introduction

The elimination of waste products from the brain is accomplished using the glymphatic system, which is a cerebrospinal fluid (CSF) transport mechanism (1, 2). In the glymphatic pathway, CSF in the para-arterial space in the brain moves to the brain parenchyma *via* aquaporin-4 (AQP-4) in the vascular end-feet of astrocytes (3). Subsequently, interstitial fluid (ISF) and waste products pass through the para-venous space and are ultimately excreted into the lymphatic system of the neck. Studies on the association between glymphatic system dysfunction and the pathogenesis of neurological diseases have been continuously conducted in recent years; glymphatic system dysfunction is known to be associated with various neurological diseases, such as Alzheimer's disease, epilepsy, stroke, and normal pressure hydrocephalus (4–7).

Several methods have been tried to evaluate the function of glymphatic system, and among them, magnetic resonance imaging (MRI) is most commonly used. Tracer studies include MRI with intrathecal gadolinium-based contrast agent (GBCA) or intravenous GBCA (8, 9). This method is mainly used in animal experiments, and its use is limited in humans because it is invasive and can cause gadolinium encephalopathy (8). In addition, MRI with intravenous GBCA requires more time to track contrast. Therefore, a non-invasive, safer and more effective method to visualize glymphatic system functions is required. Another way to evaluate glymphatic system function using MRI is phase-contrast imaging (10). It has been used to visualize fluid movement in the body, based on the principle that when a spin travels along a magnetic gradient, it undergoes a phase shift. It can be used to measure the velocity of arterial blood, venous blood, and cerebral spinal fluid (CSF). However, the phase-contrast method is limited in evaluating the dynamics of ISF in the brain (10).

To overcome these limitations of the previous methods, “diffusion tensor image analysis along the perivascular space (DTI-ALPS)” using diffusion tensor imaging (DTI) has been developed. DTI is non-invasive and has the advantage of acquiring images within minutes. The DTI-ALPS method evaluates the flow of body fluids in the direction of the perivascular space by first measuring the diffusivity of each axis and substituting this value into the formula (6). Therefore, it is a means to evaluate alterations in function of glymphatic system or dynamics of interstitial fluid in the brain. A malfunction of the glymphatic system can be inferred using this method if the DTI-ALPS index is low. Recently, the DTI-ALPS method has been applied to elucidate glymphatic system dysfunction in several neurological diseases (4–7).

Chronic kidney disease (CKD) is defined as an abnormality in the function or structure of the kidneys lasting more than 3 months that affects health (11). Criteria defined as CKD include decreased glomerular filtration rate (GFR <60 ml/min/1.73 m^2^), abnormal findings in urine tests such as albuminuria, or structural abnormalities confirmed by imaging tests (11). Although there is no internationally accepted definition of early CKD, we used the term early CKD as a common concept for patients with CKD at the pre-end stage renal disease (ESRD) stage.

Patients with CKD are at an increased risk of cerebrovascular diseases, such as stroke, and cognitive impairment (12). Complications of CKD such as uremia, other metabolic abnormalities, and anemia are risk factors for cognitive decline, and chronic inflammation in patients with CKD is related to dementia (13, 14). Therefore, it can be expected that dysfunction of glymphatic system may be present in patients with CKD. The DTI-ALPS index has previously been utilized to indicate glymphatic system dysfunction in ESRD patients, and the method's feasibility for assessing glymphatic system function in ESRD patients was verified (15). However, no research has been done on the function of the glymphatic system in patients with early CKD.

The goal of this study was to compare the function of the glymphatic system in neurologically asymptomatic patients with early CKD to that of healthy controls. In individuals with CKD, we expected that glymphatic system impairment occurs before neurological problems.

## Methods

### Participants

The institutional review board approved this investigation, which was conducted prospectively in a single tertiary hospital. All participants signed a written informed consent form. Between October 2018 and March 2021, we included neurologically asymptomatic patients with early CKD. The following were the criteria for inclusion: (1) an estimated GFR (eGFR) of > 15 ml/min/1.73 m^2^ determined using the CKD-EPI Creatinine Equation; and (2) no prior history of neurological or psychiatric diseases. Patients with structural brain lesions including tumors, stroke, or traumatic brain injury, as well as cognitive impairment, were excluded from the study. We gathered clinical data on patients with early CKD, including age, sex, presence of comorbidities like hypertension or diabetes, and laboratory results.

An age- and sex-matched control group of 18 healthy subjects with no medical, neurological, or psychiatric background was also enrolled. All healthy controls had normal MRI scans of their brains with no structural defects.

### DTI acquisition and processing

For all subjects, the identical scanner was used for DTI (3.0 T, 32-channel head coil, AchievaTx, Phillips Healthcare, Best, Netherlands). It was done with 32 different diffusion directions using spin-echo single-shot echo-planar pulse sequences (TR/TE = 8,620/85 ms, FA = 90°, slice thickness = 2.25 mm, acquisition matrix = 120 120, FOV = 240 240 mm^2^, and *b*-value = 1,000 s/mm^2^).

For preprocessing of brain MRI, the DSI studio program (version 2021 May, http://dsi-studio.labsolver.org) was utilized, which featured open-source imaging, eddy current and phase distortion artifact correction, mask setup (thresholding, smoothing, and defragmentation), and reconstruction using the DTI method.

### Calculation of the diffusion tensor imaging-analysis along the perivascular space index

**Figure 1:**
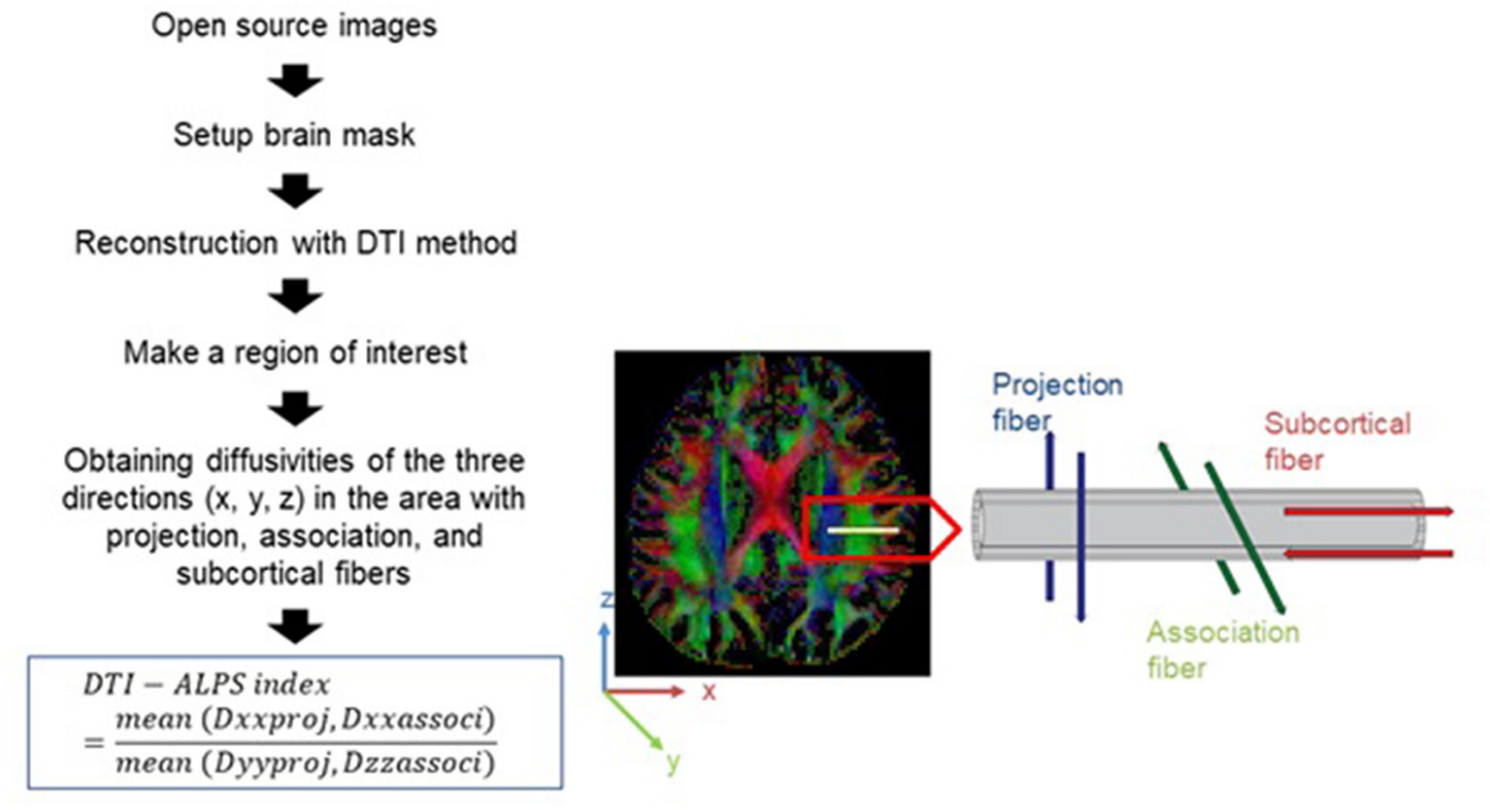
Diagram depicting how the diffusion tensor image analysis along the perivascular space (DTI-ALPS) index is calculated (16).


Figure 1 shows a flowchart of the process for calculating the DTI-ALPS index. We drew a rectangular region of interest (ROI) in which the lateral projections of the medullary veins were traced orthogonally to the primary diffusion directions. The fiber orientation and diffusivities of the three directions along the x, y, and z axes were then calculated as voxel levels in the ROI. The averaged diffusivities were obtained in each ROI, excluding the maximum and minimum values, from among the various voxels for each fiber on the same x, y, and z axes (projection, association, and subcortical fibers). The DTI-ALPS index was derived using the formula below (6, 15, 17):

$$\begin{array}{l} ALPS\;index=\frac{mean\;\left(Dxxproj,\;Dxxassoci\right)}{mean\;\left(Dyyproj,\;Dzzassoci\right)} \end{array}$$

Dxxproj: diffusivity along the x-axis in the projection fiber, Dxxassoci: diffusivity along the x-axis in the association fiber, Dyyproj: diffusivity along the y-axis in the projection fiber, Dzzassoci: diffusivity along the z-axis in the association fiber.

### Statistical analysis

The demographic and clinical characteristics and diffusivities were compared between groups using the chi-squared test or Fisher's exact test for categorical variables and Student's *t*-test for numerical variables. Categorical variables were expressed as numbers and percentages. Continuous variables with a normal distribution are represented as mean values with standard deviations. A two-tailed *p*-value of < 0.05 was used to determine statistical significance. When we conducted statistical analysis for diffusivities along the axis in the fibers, we applied multiple corrections [Bonferroni correction, *p* = 0.0055 (0.05/9)]. MedCalc^®^ Statistical Software version 20 (MedCalc Software Ltd, Ostend, Belgium; https://www.medcalc.org; 2021) was used for all statistical analyses.

## Results

### Demographics and clinical characteristics

Eighteen patients with early CKD were included in this study. Table 1 represents the clinical characteristics of patients with early CKD. Patients with early CKD were classified as having CKD stages as 3a, 3b, and 4, according to eGFR. Of the 18 patients with early CKD, eight had diabetes mellitus (DM) and thirteen had hypertension (HTN), and five of these patients had both HTN and DM. Two patients had no comorbidities.

**Table 1:** Clinical characteristics of the patients with early CKD.

**Clinical data**	**Patients with early CKD (*N* = 18)**	**Healthy controls (*N* = 18)**	***p*-value**
**Demographic data**			
Age, years (SD)	65.9 (9.9)	66.4 (6.3)	0.842
Male, *N* (%)	9 (50.5)	11 (61.1)	0.508
**Comorbidities**			
Diabetes mellitus, *N* (%)	8 (44.4)		
Hypertension, *N* (%)	13 (72.2)		
CKD stage			
3a	5 (27.8)		
3b	10 (55.6)		
4	3 (16.7)		
**Laboratory data**			
Hemoglobin, g/dL (SD)	11.9 (1.9)		
Hematocrit, % (SD)	35.7 (5.5)		
eGFR, ml/min/1.73 m^2^ (SD)	39.7 (9.4)		
Protein, g/dL (SD)	7.2 (0.6)		
Albumin, g/dL (SD)	4.0 (0.4)		
Aspartate aminotransferase, U/L (SD)	22.4 (6.6)		
Alanine aminotransferase, U/L (SD)	18.8 (8.2)		
BUN, mg/dL (SD)	26.0 (6.4)		
Creatinine, mg/dL (SD)	1.7 (0.4)		
Sodium, mmol/L (SD)	140.9 (2.6)		
Potassium, mmol/L (SD)	4.7 (0.5)		
Chloride, mmol/L (SD)	106.1 (4.0)		
Calcium, mg/dL (SD)	8.4 (1.2)		
Phosphate, mg/dL (SD)	3.6 (0.5)		
Total CO_2_ contents, mmol/L (SD)	23.9 (3.0)		
Total cholesterol, , mg/dL (SD)	153.1(35.1)		

CKD, chronic kidney disease; SD, standard deviations; eGFR, estimated GFR.

### Diffusivities along the axis in the fibers

Between patients with early CKD and healthy controls, there was significant alteration in diffusivity along the y-axis in projection fiber. The diffusivities in other x, y, and z axes in projection, association and subcortical fibers had no significant alteration in early CKD patients (Table 2). In addition, diffusivities were not significantly different in early CKD patients depending on DM or HTN, respectively (Table 3).

**Table 2:** The differences of the diffusivities along the axis in the fibers between patients with early CKD and healthy controls.

	**Patients with early CKD (*****N*** = **18)**		**Healthy controls (*****N*** = **18)**		
	**Mean**	**SD**	**Mean**	**SD**	***p*-value**
**Projection fiber**					
Dxx	0.0006	0.0001	0.0006	0.0001	0.2603
Dyy	0.0006	0.0001	0.0005	0.0001	0.0046
Dzz	0.0011	0.0001	0.0010	0.0001	0.0540
**Association fiber**					
Dxx	0.0007	0.0001	0.0007	0.0001	0.7393
Dyy	0.0011	0.0001	0.0010	0.0001	0.1290
Dzz	0.0005	0.0001	0.0004	0.0001	0.0101
**Subcortical fiber**					
Dxx	0.0011	0.0001	0.0011	0.0001	0.7976
Dyy	0.0007	0.0001	0.0007	0.0001	0.7833
Dzz	0.0006	0.0002	0.0006	0.0001	0.6433
DTI-ALPS index	1.2594	0.1994	1.4777	0.2327	0.0048

CKD, chronic kidney disease; SD, standard deviation; Dxx, diffusivity along the x-axis; Dyy, diffusivity along the y-axis; Dzz, diffusivity along the z-axis; DTI-ALPS index, diffusion tensor image analysis with the perivascular space index.

**Table 3:** The differences of the diffusivities along the axis in the fibers between early CKD patients with or without comorbidities.

	**Early CKD patients** **with DM (*****N*** = **8)**		**Early CKD patients without DM (*****N*** = **10)**		
	**Mean**	**SD**	**Mean**	**SD**	***p*-value**
**Projection fiber**					
Dxx	0.0006	0.0001	0.0007	0.0001	0.0747
Dyy	0.0006	0.0002	0.0006	0.0001	0.5264
Dzz	0.0011	0.0002	0.0010	0.0001	0.9049
**Association fiber**					
Dxx	0.0007	0.0001	0.0007	0.0001	0.3734
Dyy	0.0011	0.0001	0.0011	0.0001	0.7157
Dzz	0.0004	0.0001	0.0005	0.0001	0.5796
**Subcortical fiber**					
Dxx	0.0011	0.0001	0.0011	0.0001	0.8137
Dyy	0.0007	0.0001	0.0007	0.0002	0.8667
Dzz	0.0006	0.0001	0.0007	0.0002	0.5735
DTI-ALPS index	1.2004	0.2321	1.3067	0.1662	0.2738
	**Early CKD patients with HTN (*****N*** **=** **13)**		**Early CKD patients without HTN(*****N*** **=** **5)**		
	**Mean**	**SD**	**Mean**	**SD**	***p*** **-value**
**Projection fiber**					
Dxx	0.0006	0.0001	0.0006	0.0001	0.1235
Dyy	0.0006	0.0002	0.0006	0.0001	0.9110
Dzz	0.0011	0.0001	0.0010	0.0002	0.5258
**Association fiber**					
Dxx	0.0007	0.0001	0.0006	0.0001	0.4225
Dyy	0.0011	0.0001	0.0011	0.0001	0.8597
Dzz	0.0005	0.0001	0.0005	0.0001	0.9065
**Subcortical fiber**					
Dxx	0.0011	0.0001	0.0010	0.0001	0.4481
Dyy	0.0007	0.0002	0.0007	0.0001	0.6660
Dzz	0.0006	0.0001	0.0007	0.0002	0.1136
DTI-ALPS index	1.2887	0.1999	1.1834	0.1978	0.3307

CKD, chronic kidney disease; DM, diabetic mellitus; SD, standard deviation; Dxx, diffusivity along the x-axis; Dyy, diffusivity along the y-axis; Dzz, diffusivity along the z-axis; DTI-ALPS index, diffusion tensor image analysis with the perivascular space index; HTN, hypertension.

### Diffusion tensor imaging-analysis along the perivascular space index

The DTI-ALPS index showed a significant difference between patients with early CKD and healthy controls. Patients with early CKD had a lower DTI-ALPS index than healthy controls (1.259 ± 0.199 vs. 1.477 ± 0.232, *p* = 0.004) (Table 2). The DTI-ALPS index was not significantly different in early CKD patients depending on DM or HTN, respectively (Table 3).

In the correlation analysis, the DTI-ALPS index had no significant relationship with other clinical factors, such as age (*r* = −0.202, *p* = 0.422), stage of CKD (*r* = −0.006, *p* = 0.980), and other laboratory data (Hemoglobin, *r* = 0.314, *p* = 0.205; Hematocrit, *r* = 0.280, *p* = 0.261; eGFR, *r* = 0.129, *p* = 0.610; Albumin, *r* = 0.413, *p* = 0.089; serum protein level, *r* = −0.403, *p* = 0.097, Aspartate aminotransferase, *r* = 0.154, *p* = 0.542; Alanine aminotransferase, *r* = 0.376, *p* = 0.124; BUN, *r* = −0.032, *p* = 0.899; Creatinine, *r* = −0.108, *p* = 0.669; Sodium, *r* = −0.170, *p* = 0.500; Potassium, *r* = 0.051, *p* = 0.840; Chloride, *r* = 0.062, *p* = 0.808; Calcium, *r* = −0.298, *p* = 0.230; Phosphate, *r* = 0.034, *p* = 0.894; Total CO_2_ contents, *r* = −0.147, *p* = 0.560).

## Discussion

First, we compared glymphatic system dysfunction between neurologically asymptomatic patients with early CKD and healthy controls *via* the DTI-ALPS method. The study's key conclusion was that the DTI-ALPS index was significantly lower in patients with early CKD than in healthy controls, which suggested that dysfunction of glymphatic system was present in patients with early CKD. In the correlation analysis, the DTI-ALPS index had no significant relationship with other clinical factors.

As previously mentioned, The DTI-ALPS method is one that can be utilized in order to do an evaluation of the diffusivity in the perivascular area. It is expected that the ratio of the x-axis diffusivity of the projection fibers and the area of the association fibers (Dxproj and Dxassoc) to the diffusivity perpendicular to them (Dyproj and Dzassoc) express the influence of water diffusion along the perivascular space, which reflects the activity of the glymphatic system in the individual cases (6). A higher ratio in the DTI-ALPS method indicates more water diffusivity along the perivascular space. Therefore, a significantly lower DTI-ALPS index in early CKD patients than in healthy controls suggests dysfunction of the glymphatic system in early CKD patients.

Neurological problems in patients with CKD are likely to originate from multifactorial factors (18). As mentioned above, cerebrovascular disease, uremic toxins, other metabolic abnormalities, anemia, and chronic inflammation in patients with CKD are the causes of neurologic complications in CKD. Pathophysiology may be divided into vascular and neurodegenerative mechanisms. In particular, neurodegenerative mechanisms may be associated with the low efficiency of elimination of metabolic waste or uremic toxins, including uric acid, p-cresyl sulfate, indoxyl sulfate, tumor necrosis factor-α, interleukin-1β and interleukin-6 in patients with CKD (19). In this study, glymphatic system dysfunction was suggested even in patients with early CKD without neurological problems. In addition, alterations of structural connectivity, accounting for information processing of neural networks, in the brains of patients with early CKD have also been observed in our previous study (20). These results suggest that alterations in structural connectivity begin relatively earlier than functional alterations in CKD patients. Dysfunction of glymphatic system may also be associated to alterations in the brain connectivity.

The exact mechanism of glymphatic dysfunction in patients with early CKD has not yet been elucidated. Two assumptions were made to explain the results of this study. In the glymphatic system, Glymphatic inflow is thought to be influenced by arterial pulsation (21). Risk factors such as DM, HTN, hypercoagulable state, chronic inflammation, oxidative stress, and uremic toxin in patients with CKD induce vascular injury and endothelial dysfunction (22, 23). Arterial stiffening and reduction in cerebral blood flow induced by vascular damage are thought to reduce glymphatic influx and may cause dysfunction of glymphatic system in patients with CKD. Another theory that may explain dysfunction of glymphatic system in patients with CKD is its association with aquaporins (AQPs). AQPs are a class of selective transmembrane channels that are responsible for transporting mostly water and, in some subtypes, low molecular weight solutes across the membranes of cells. According to research conducted on animals, AQPs have been linked to both acute kidney damage and a variety of CKDs, such as polycystic kidney disease, diabetic nephropathy, and renal cell carcinoma (24). In particular, a previous study showed of decreased kidney AQP4 expression in mice with hydronephrosis (25). Therefore, although studies on AQP-4 are insufficient, patients with early CKD may develop glymphatic system dysfunction due to decreased AQP-4 expression, which plays an important factor in the glymphatic pathway. For the above two reasons, there is dysfunction of glymphatic system in patients with CKD, which may be one of the pathophysiological mechanisms of neurological problems in patients with early CKD.

In this study, we found that DTI-ALPS index is significantly decreased in patients with early CKD, which allow us to infer that there is a glymphatic dysfunction in early CKD. However, it is well known that the main causes of CKD are DM and HTN, and each independently affect glymphatic function in previous studies (21, 26). DM and HTN induce both micro- and macro-vascular damage which increase the risk of developing small vessel disease with enlargement of the perivascular space. In addition, vascular inflammation may also play an important role in the enlargement of the perivascular space (27, 28). Recent research has indicated that carotid atherosclerosis may reduce the DTI-ALPS index and impair glymphatic system function (29). Thus, we performed analysis according to the comorbidities, requiring caution in determining whether the results of our research are due to DM or HTN or to Early CKD itself. There was no significant difference between diffusivities and DTI-ALPS index in the patient group according to DM or HTN. Nevertheless, there is a limitation in interpretation because the sample size was small and there were only two patients without both DM and HTN.

Previous studies have shown a decrease in glymphatic activity during aging (30, 31). The glymphatic system dysfunction in the elderly is associated with a decrease in CSF influx and a reduction in metabolites clearance (31). Reactive gliosis, loss of perivascular AQP4 polarization, and arterial wall stiffening reduce arterial pulsation as aging advances (1, 31–33). In this study, there was no correlation between the DTI-ALPS index and age in patients with early CKD, inconsistent with the results of previous studies, which may be mainly due to the small number of samples enrolled into the study.

This is the first study to investigate the function of the glymphatic system in patients with early CKD, and we were able to show that the glymphatic system is dysfunctional in patients with early CKD compared to healthy controls. However, this study has several limitations. First, our study‘s sample size is small. It is not easy to recommend DTI imaging for enrollment of patients with early CKD. Second, although patients with early CKD who did not undergo dialysis were included, those with CKD stages 3 and 4 were also enrolled. Further studies on glymphatic system dysfunction at each stage, including a larger number of patients, are required. Third, patients who had ischemic stroke were excluded, but cerebral artery conditions such as arteriosclerosis was not considered. MR angiography was not performed on patients and only total cholesterol was included in the lipid profile. It will be necessary to evaluate the DTI-ALPS index according to the degree of the atherosclerosis while tracking the patients with early CKD. Lastly, although we excluded patients with structural brain lesions or previous history of neurological disorders, we could not control the white matter hyper-intensity burden between patients with early CKD and healthy controls. For this reason, the difference in diffusivity along the y axis in projection fiber has been identified. And it may also be due to the small sample size.

## Conclusion

Using the DTI-ALPS index, we first identified glymphatic system dysfunction in patients with early CKD. This may be related to the pathophysiology of neurological problems, including cognitive impairment, in patients with early CKD. This study also provides opportunities for advances in the pathophysiology and treatment of patients with CKD that can be used in future studies.

## Data availability statement

The raw data supporting the conclusions of this article will be made available by the authors, without undue reservation.

## Ethics statement

The studies involving human participants were reviewed and approved by Institutional Review Board of Haeundae Paik hospital. The patients/participants provided their written informed consent to participate in this study.

## Author contributions

CH and DL participated in data collection, data interpretation, and manuscript writing. KP participated in data collection, data interpretation, and report writing. YL, SP, YK, JK, and BY participated in data collection and data interpretation. BP supervised analysis, participated in study design, data interpretation, and manuscript writing. All authors provided critical feedback, read, and approved the final manuscript.

## Conflict of interest

Author BY was employed by DEEPNOID.

The remaining authors declare that the research was conducted in the absence of any commercial or financial relationships that could be construed as a potential conflict of interest.

## Publisher's note

All claims expressed in this article are solely those of the authors and do not necessarily represent those of their affiliated organizations, or those of the publisher, the editors and the reviewers. Any product that may be evaluated in this article, or claim that may be made by its manufacturer, is not guaranteed or endorsed by the publisher.

## References

[1] Iliff JJ Wang M Liao Y Plogg BA Peng W Gundersen GA. A paravascular pathway facilitates CSF flow through the brain parenchyma and the clearance of interstitial solutes, including amyloid β. Sci Transl Med. (2012) 4:147ra11. 10.1126/scitranslmed.3003748 22896675 PMC3551275

[2] Rasmussen MK Mestre H Nedergaard M. The glymphatic pathway in neurological disorders. Lancet Neurol. (2018) 17:1016–24. 10.1016/S1474-4422(18)30318-1 30353860 PMC6261373

[3] Nagelhus EA Ottersen OP. Physiological roles of aquaporin-4 in brain. Physiol Rev. (2013) 93:1543–62. 10.1152/physrev.00011.2013 24137016 PMC3858210

[4] Lee DA Park BS Ko J Park SH Lee YJ Kim IH. Glymphatic system dysfunction in temporal lobe epilepsy patients with hippocampal sclerosis. Epilepsia Open. (2022) 7:306–14. 10.1002/epi4.12594 35305294 PMC9159256

[5] Bae YJ Choi BS Kim JM Choi JH Cho SJ Kim JH. Altered glymphatic system in idiopathic normal pressure hydrocephalus. Parkinsonism Relat Disord. (2021) 82:56–60. 10.1016/j.parkreldis.2020.11.009 33248394

[6] Taoka T Masutani Y Kawai H Nakane T Matsuoka K Yasuno F. Evaluation of glymphatic system activity with the diffusion MR technique: diffusion tensor image analysis along the perivascular space (DTI-ALPS) in Alzheimer's disease cases. Jpn J Radiol. (2017) 35:172–8. 10.1007/s11604-017-0617-z 28197821

[7] Toh CH Siow TY. Glymphatic dysfunction in patients with ischemic stroke. Front Aging Neurosci. (2021) 13:756249. 10.3389/fnagi.2021.756249 34819849 PMC8606520

[8] Arlt S Cepek L Rustenbeck HH Prange H Reimers CD. Gadolinium encephalopathy due to accidental intrathecal administration of gadopentetate dimeglumine. J Neurol. (2007) 254:810–2. 10.1007/s00415-006-0439-x 17401744

[9] Iliff JJ Lee H Yu M Feng T Logan J Nedergaard M. Brain-wide pathway for waste clearance captured by contrast-enhanced MRI. J Clin Invest. (2013) 123:1299–309. 10.1172/JCI67677 23434588 PMC3582150

[10] Taoka T Naganawa S. Glymphatic imaging using MRI. J Magn Reson Imaging. (2020) 51:11–24. 10.1002/jmri.26892 31423710

[11] Stevens PE Levin A. Evaluation and management of chronic kidney disease: synopsis of the kidney disease: improving global outcomes 2012 clinical practice guideline. Ann Intern Med. (2013) 158:825–30. 10.7326/0003-4819-158-11-201306040-00007 23732715

[12] Kurella Tamura M Wadley V Yaffe K McClure LA Howard G Go R. Kidney function and cognitive impairment in US adults: the Reasons for Geographic and Racial Differences in Stroke (REGARDS) Study. Am J Kidney Dis. (2008) 52:227–34. 10.1053/j.ajkd.2008.05.004 18585836 PMC2593146

[13] Wood JA Wood PL Ryan R Graff-Radford NR Pilapil C Robitaille Y. Cytokine indices in Alzheimer's temporal cortex: no changes in mature IL-1 beta or IL-1RA but increases in the associated acute phase proteins IL-6, alpha 2-macroglobulin and C-reactive protein. Brain Res. (1993) 629:245–52. 10.1016/0006-8993(93)91327-O 7509248

[14] Kurella M Yaffe K Shlipak MG Wenger NK Chertow GM. Chronic kidney disease and cognitive impairment in menopausal women. Am J Kidney Dis. (2005) 45:66–76. 10.1053/j.ajkd.2004.08.044 15696445

[15] Heo CM Lee WH Park BS Lee YJ Park S Kim YW. Glymphatic dysfunction in patients with end-stage renal disease. Front Neurol. (2021) 12:809438. 10.3389/fneur.2021.809438 35145471 PMC8821099

[16] Lee DA Lee HJ Park KM. Glymphatic dysfunction in isolated REM sleep behavior disorder. Acta Neurol Scand. (2022) 145:464–70. 10.1111/ane.13573 34918348

[17] Taoka T Ito R Nakamichi R Kamagata K Sakai M Kawai H. Reproducibility of diffusion tensor image analysis along the perivascular space (DTI-ALPS) for evaluating interstitial fluid diffusivity and glymphatic function: CHanges in Alps index on Multiple conditiON acquIsition eXperiment (CHAMONIX) study. Jpn J Radiol. (2022) 40:147–58. 10.1007/s11604-021-01187-5 34390452 PMC8803717

[18] Arnold R Issar T Krishnan AV Pussell BA. Neurological complications in chronic kidney disease. JRSM Cardiovasc Dis. (2016) 5:2048004016677687. 10.1177/2048004016677687 27867500 PMC5102165

[19] Watanabe K Watanabe T Nakayama M. Cerebro-renal interactions: impact of uremic toxins on cognitive function. Neurotoxicology. (2014) 44:184–93. 10.1016/j.neuro.2014.06.014 25003961

[20] Lee YJ Yoon E Park S Kim YW Kim SE Ko J. Alteration of brain connectivity in neurologically asymptomatic patients with chronic kidney disease. Medicine. (2021) 100:e25633. 10.1097/MD.0000000000025633 33879740 PMC8078245

[21] Mortensen KN Sanggaard S Mestre H Lee H Kostrikov S Xavier ALR. Impaired glymphatic transport in spontaneously hypertensive rats. J Neurosci. (2019) 39:6365–77. 10.1523/JNEUROSCI.1974-18.2019 31209176 PMC6687896

[22] Madero M Gul A Sarnak MJ. Cognitive function in chronic kidney disease. Semin Dial. (2008) 21:29–37. 10.1111/j.1525-139X.2007.00384.x 18251955

[23] Stinghen AE Pecoits-Filho R. Vascular damage in kidney disease: beyond hypertension. Int J Hypertens. (2011) 2011:232683. 10.4061/2011/232683 21876786 PMC3160729

[24] He J Yang B. Aquaporins in renal diseases. Int J Mol Sci. (2019) 20:366. 10.3390/ijms20020366 30654539 PMC6359174

[25] Ampawong S Klincomhum A Likitsuntonwong W Singha O Ketjareon T Panavechkijkul Y. Expression of aquaporin-1,−2 and−4 in mice with a spontaneous mutation leading to hydronephrosis. J Comp Pathol. (2012) 146:332–7. 10.1016/j.jcpa.2011.08.005 21945302

[26] Jiang Q Zhang L Ding G Davoodi-Bojd E Li Q Li L. Impairment of the glymphatic system after diabetes. J Cereb Blood Flow Metab. (2017) 37:1326–37. 10.1177/0271678X16654702 27306755 PMC5453454

[27] Wardlaw JM Smith C Dichgans M. Mechanisms of sporadic cerebral small vessel disease: insights from neuroimaging. Lancet Neurol. (2013) 12:483–97. 10.1016/S1474-4422(13)70060-7 23602162 PMC3836247

[28] Doubal FN MacLullich AM Ferguson KJ Dennis MS Wardlaw JM. Enlarged perivascular spaces on MRI are a feature of cerebral small vessel disease. Stroke. (2010) 41:450–4. 10.1161/STROKEAHA.109.564914 20056930

[29] Liu H Yang S He W Liu X Sun S Wang S. Associations among diffusion tensor image along the perivascular space (DTI-ALPS), enlarged perivascular space (ePVS), and cognitive functions in asymptomatic patients with carotid plaque. Front Neurol. (2021) 12:789918. 10.3389/fneur.2021.789918 35082748 PMC8785797

[30] Jessen NA Munk ASF Lundgaard I Nedergaard M. The glymphatic system: a beginner's guide. Neurochem Res. (2015) 40:2583–99. 10.1007/s11064-015-1581-6 25947369 PMC4636982

[31] Kress BT Iliff JJ Xia M Wang M Wei HS Zeppenfeld D. Impairment of paravascular clearance pathways in the aging brain. Ann Neurol. (2014) 76:845–61. 10.1002/ana.24271 25204284 PMC4245362

[32] Sabbatini M Barili P Bronzetti E Zaccheo D Amenta F. Age-related changes of glial fibrillary acidic protein immunoreactive astrocytes in the rat cerebellar cortex. Mech Ageing Dev. (1999) 108:165–72. 10.1016/S0047-6374(99)00008-1 10400309

[33] Iliff JJ Wang M Zeppenfeld DM Venkataraman A Plog BA Liao Y. Cerebral arterial pulsation drives paravascular CSF–interstitial fluid exchange in the murine brain. J Neurosci. (2013) 33:18190–9. 10.1523/JNEUROSCI.1592-13.2013 24227727 PMC3866416

